# Coding traumatic brain injury with the abbreviated injury scale following a standardised radiologic template will improve classification of trauma populations

**DOI:** 10.1007/s00330-025-11384-9

**Published:** 2025-01-31

**Authors:** Jan C. van Ditshuizen, Menco J. S. Niemeyer, Esther M. M. Van Lieshout, Dennis Den Hartog, Jacob J. Visser, Karlijn J. P. van Wessem, Michiel H. J. Verhofstad

**Affiliations:** 1 Trauma Research Unit, Department of Surgery, Erasmus MC, University Medical Center Rotterdam, Rotterdam, The Netherlands; 2 Trauma Centre Southwest Netherlands, Erasmus MC, University Medical Center Rotterdam, Rotterdam, The Netherlands; 3 Department of Trauma Surgery, University Medical Center Utrecht, Utrecht, The Netherlands; 4 Department of Radiology & Nuclear Medicine, Erasmus MC, University Medical Center Rotterdam, Rotterdam, The Netherlands

**Keywords:** Injury Scale, abbreviated, Coding, clinical, Injury, Registries, Improvement, quality

## Abstract

**Introduction:**

Injury coding with the Abbreviated Injury Scale (AIS) is an important element for benchmarking, trauma registries and research.

**Objective:**

To compare the severity of traumatic brain injury (TBI) coding derived from the AIS with or without the use of a standardised radiologic template.

**Methods:**

A retrospective two-centre cohort study including patients aged ≥ 18 years with isolated TBI admitted to an intensive care between 2011 and 2016 was conducted. TBI was re-coded to conform the AIS by coders, and CT-brain imaging was reassessed by a neuro-radiologist following a standardised radiologic template from which AIS codes were derived.

**Results:**

A total of 560 patients were included (median age 57, 37% female). The percentage of MAIS ≥ 4 and major trauma was higher when AIS coding for TBI was derived from a standardised radiologic template vs. coding without (*n* = 456 (81.4%) and *n* = 374 (66.8%), *p* < 0.001; *n* = 441 (78.8%) and *n* = 352 (62.9%), *p* < 0.001, respectively). There was an inter-centre difference in the proportion of MAIS ≥ 4 re-coded without a standardised radiologic template (*n* = 212 (68.2%) and *n* = 140 (56.2%), *p* = 0.004), and no difference when re-coded with the template (*n* = 251 (80.7%) and *n* = 190 (76.3%), *p* = 0.206).

**Conclusion:**

Coding TBI with AIS based on a standardised radiologic template results in fewer missed AIS head codes, more detailed AIS head codes, and more patients classified as ‘major trauma’.

**Key Points:**

***Question*** *Radiologic reports are an important source for injury coding with the abbreviated injury scale (AIS) and are often not sufficiently specific*.

***Findings*** *An AIS-based standardised radiologic template for reporting resulted in more detailed AIS head codes and more patients classified as major trauma*.

***Clinical relevance*** *Injury coding with the AIS based on a standardised radiologic template will improve exchanging medical information in the acute health care setting and classification of trauma populations*.

## Introduction

Injury coding is an important element for quality benchmarking of trauma care and identification and selection of patient groups within trauma registries [1–5]. Injury coding is mostly done retrospectively by coders based on extensive information in electronic health records (EHR), that builds up throughout the acute health care chain. Much information—such as blood loss, open fractures, or coma—is estimated semi-quantitatively or qualitatively described in electronic health records (EHR). The written clinical and radiological information is the main source for injury coders. However, a correct interpretation may require expertise. To facilitate an efficient coding process and optimise reliability of the codes registered, omission of relevant information in EHRs about injuries should be avoided.

The Abbreviated Injury Scale (AIS) details and classifies traumatic injuries according to a very specific code structure [6–8]. Each AIS injury code is assigned a suffix, reflecting the severity of a specific injury, from minor severity to untreatable. These discrete severity levels we developed in the context of 30-days mortality—not permanent disability. Because medical care and treatment opportunities improve over time, mortality decreases in general. Therefore, these severity levels are periodically recalculated for specific AIS codes [9–13]. The associated Injury Severity Score (ISS) is derived from the AIS coded injuries in the various body areas within a single individual. An ISS > 15 is a threshold for ‘major trauma’ worldwide [14–16]. Although the AIS and subsequent ISS are used in individual patients to assess the a priori risk to die and on a population level to measure quality of trauma care, both tools are subject to methodological and practical limitations [17]. The main causes of death after trauma—at least in the western world—are major bleeding and traumatic brain injury.

Uniform injury coding is essential to achieve reliable source data, not only to compare individual patients but also to correct for case-mix when comparing quality of care between trauma centres. The educational background of coders is diverse, ranging from administrative medical personnel to experienced doctors. Interpretation of extensive medical information on injuries is mostly reserved for clinicians with medical experience [18–21]. Various studies have shown variability between injury coders and between trauma facilities in injury coding with the AIS from its developing years [19, 22–24]. Especially injury coding of TBI has shown significant variability [19, 25].

The abovementioned factors raise questions on correctness of benchmarking, quality indicators, and (sub)population analysis that are based on certain injuries or classified with a specific ISS threshold. Radiologic reports are an important source for injury coding. An important element that could attribute to quality improvement is to minimise the number of inconclusive radiology reports. Inconclusive radiologic reports are those in which the reporting is not specific enough for accurate AIS coding, leading to an over-reliance on unsubstantiated or conservative injury coding. Additional modalities in radiologic reporting could potentially positively impact injury coding, thus classification of trauma populations following their injuries, and improve registry data analysis.

A standardised radiologic reporting template for Computed Tomography (CT) scans on head compatible with the AIS was developed with the intention of streamlining clinical injury coding. The lack of uniformity of injury reporting in electronic health records was hypothesised to negatively impact classifying trauma populations by injury severity according to the AIS. This study aimed to measure to what extent a standardised radiologic template influences AIS coding and severity assessment of traumatic brain injuries.

## Methods

The local Medical Research Ethics Committee concluded this study was not subject of the Medical Research Involving Human Subjects Act (MEC-2022-0779) and provided a waiver of consent. Data are reported in line with strengthening the reporting of observational studies in epidemiology (STROBE) [26].

### Study setting

Both participating level I trauma centres are located in urbanised and industrialised areas. These centres have a regional role in the admission of major trauma patients from their respective trauma regions, and both have neurosurgical facilities around the clock. Serving multiple emergency medical services and having the availability of a physician-staffed helicopter, their combined catchment area encompasses 3.5 million inhabitants.

### Study design

This two-centre retrospective cohort study was conducted on a previously collected dataset, and inclusion and exclusion criteria were taken from the corresponding study [27]. Only ICU patients were selected to definitively exclude cases of minor TBI, to address potential issues of over-triage or overestimation of the AIS score, and to partially mitigate the impact of inter-observer variability. Trauma patients aged ≥ 18 years who sustained moderate-to-severe TBI with a maximum AIS (MAIS) of the head ≥ 3 and MAIS ≤ 2 in other anatomical regions admitted to an intensive care unit via the emergency room (ER) from January 1, 2011, until December 31, 2016, were included. Patients were excluded when admitted with either an isolated cervical spinal injury, with the presence of a non-traumatic intracranial haematoma, or when secondary brain damage was caused due to asphyxia.

### Data collection—baseline

Patients were selected from the Dutch Nationwide Trauma Registry (DNTR) of the two participating level I trauma centres [28]. Demographics, clinical outcome measures and in-hospital mortality data were extracted from the DNTR of both centres in December 2017 (age, sex, hospital and ICU length of stay, trauma mechanism, AIS with derived MAIS and ISS, and in-hospital mortality).

### Data collection—image reporting

A standardised radiologic template for reporting traumatic brain injuries was developed. This template was based on most elements of the AIS revision 2005 (update 2008) head section (type of vault/base fracture and clinical elements were left out of the template), the Marshall classification of TBI, and the Rotterdam score [6, 29, 30]. The following elements were represented in the template: skull base fracture, skull vault fracture, pneumocephalus, subarachnoid bleeding, epidural bleeding (mm/cc), subdural bleeding (mm/cc), cerebral contusion (mm), petechial bleeding, midline shift (mm), herniation, compressed basal cisterns, third ventricle compression, and remarks (Supplementary Fig. 1).

All acute phase CT-brain imaging was reassessed by two neuro-radiologists in each respective centre in July 2019. All four radiologists (J.D.K., J.W.D., M.V.H., L.C.) were fellow and had five to six years of experience in reporting CT scans. Reassessments were conducted using a standardised radiologic template.

### Data collection—injury coding

From the reassessment of the radiologists, each TBI patient was AIS coded, the severity per injury (MAIS head) was derived, and ISS was calculated, termed “template coding”.

Each participating centre provided a person who assigned injury codes to each TBI patient, i.e., a coder for recoding. In April 2021, traumatic brain injuries were re-coded, alternating by the coder of the other centre using the AIS revision 2005 (update 2008) [6]. Both coders (B.S., J.V.D.) had 10 years of experience with AIS injury coding. In the country where the study took place, all radiologic (TBI) reports are supervised by a senior (neuro-)radiologist. For each TBI patient MAIS head was derived from AIS codes, and the ISS was calculated. In addition, the number of ‘not further specified’ AIS codes was determined. Not further specified AIS codes occur when detailed information is lacking on the type of injury or the severity of an injury.

**Fig. 1:**
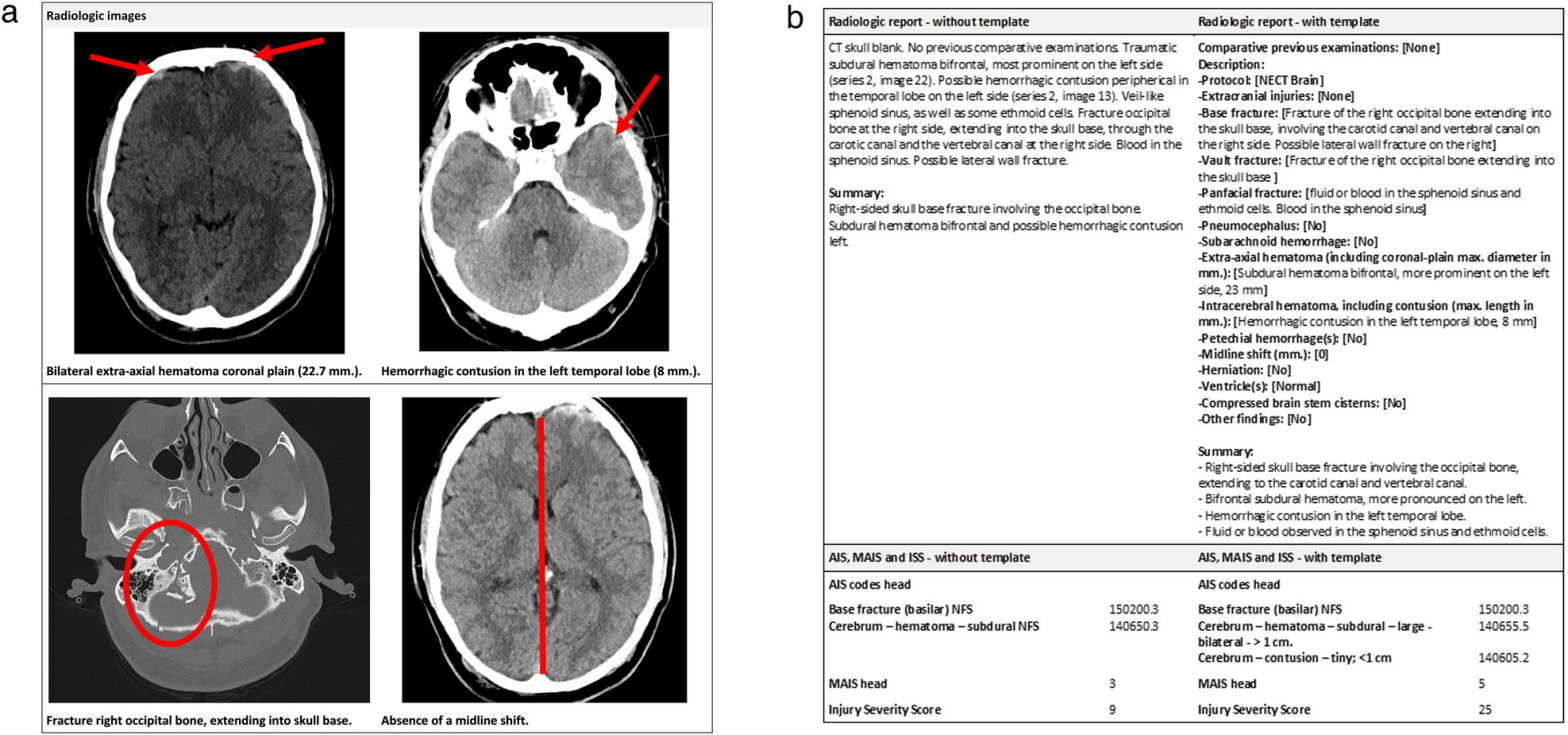
**a** Example of radiologic images with visible traumatic brain injuries. **b** Example of AIS injury coding without and with a standardised radiologic template based on images of **a**

Figure 1a and 1b combined illustrate how AIS codes were assigned to a TBI patient by radiologic reports without and with a standardised radiologic template. In Fig. 1a, four images from the same CT scan taken from a TBI patient are displayed. In Fig. 1b, a radiologic report without the template is shown (left), and a radiologic report with the template is shown (right), as well as AIS codes, derived MAIS head, and calculated ISS based on each radiologic report.

### Data collection—outcome

Severity of TBI was defined per MAIS, and maximum AIS was dichotomised in MAIS = 3 and MAIS ≥ 4. ‘Major trauma’ was defined as ISS > 15. The number of ‘Not further specified’ (NFS) AIS head codes were derived per patient.

### Statistical analysis

Statistical analyses were done with Statistical Package for Social Sciences version 25.0 (SPSS).

Frequencies and percentages are given for categorical variables, and continuous variables are displayed as median (P_25_-P_75_) since continuous data were non-parametric based on the Shapiro–Wilk test. Differences in baseline characteristics between the two participating centres were tested using Mann–Whitney U-tests for comparing two groups, and for categorical variables, χ^2^-tests were done.

For comparing dichotomised MAIS head between recoding without and with a standardised radiologic template, the false positive rates and false negative rates were calculated and McNemar tests were done. The false positive rate was defined as an overestimation of the injury severity by the (re)coders, and the false negative rate was defined as an under-estimation of the injury severity by the (re)coders, both relative to the injury severity coded based on the standardised radiologic template. The true positive rate, or sensitivity, was defined as the proportion classified as MAIS < 4 by recoding without a standardised radiologic template of the group classified as MAIS < 4 re-coded with a standardised radiologic template. The true negative rate, or specificity, was defined as the proportion classified as MAIS ≥ 4 by recoding without a standardised radiologic template of the group classified as MAIS ≥ 4 re-coded with a standardised radiologic template. A comparison between ISS derived from regular AIS coding and ISS derived from AIS coding by template was done with the intraclass correlation coefficient (ICC). The same was done for the comparison between ISS extracted from the DNTR and ISS derived from AIS coding by template as a sensitivity analysis and only done for the period 2015–2016 since the ISS extracted from the DNTR was derived from AIS98 until 2014 and from AIS08 in 2015 and 2016.

The ICC ranges from 0 (no reliability) to 1 (perfect reliability) [31]. The ICC absolute agreement for injury severity classification with and without a structured radiologic template was tested and presented with Pearson’s *r* and a 95% confidence interval (CI). A two-way mixed model was chosen because the coders and radiologists were non-randomly assigned to a sample. The classification of Landis and Koch was used to classify agreement between ISS derived from re-coded AIS without and with a standardised radiologic template (0.00–0.20, Slight, 0.20–0.40, Fair 0.40–0.60, Moderate 0.61–0.80, Substantial, 0.81–1.00 Almost perfect) [32]. In addition, Bland–Altman plots were created, plotting the differences between the ISS calculated following two methods (recoding and radiologic template, or DNTR extracted ISS and radiologic template on the *y*-axis and the mean of the two methods on the *x*-axis) [31]. Data are shown for the total cohort and for the individual hospitals. All statistical tests were two-sided. A *p*-value of < 0.05 was considered significant.

## Results

**Fig. 2:**
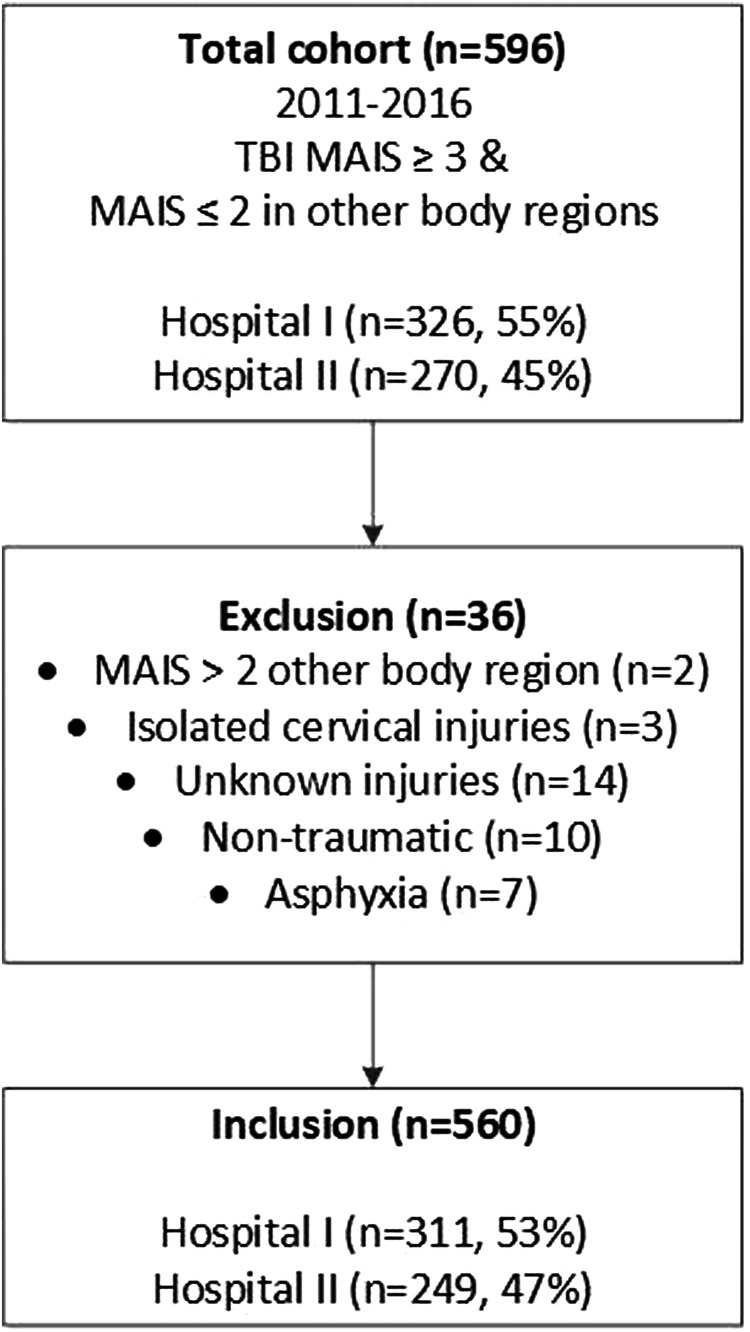
Study flow chart

A total of 596 patients were selected, of whom 36 were excluded because of either an isolated cervical spinal injury, the presence of a non-traumatic intracranial haematoma, or when secondary brain damage was caused due to asphyxia, remaining with a final dataset of 560 included patient records; Hospital I included 311 (53%) patients and hospital II included 249 (47%) patients (Fig. 2).

### Study population

The study populations across the hospitals did not differ significantly in terms of age, sex, hospital stay, ICU admission, or mortality (Table 1). There was a significant difference in ISS (25 P_25_-P_75_ 20–27 vs. 21 P_25_-P_75_ 17–26, *p* < 0.001) and mechanisms of injury (*p* < 0.001) between both populations. Hospital I had more high energy falls and non-accidental trauma (*n* = 46, 14.8% and *n* = 33, 10.6%, respectively), while hospital II had more falls from stairs and road traffic accidents (*n* = 57 (18.3%) and *n* = 128 (41.2%), respectively).

**Table 1:** Characteristics of the total cohort and epidemiological comparison between both participating hospitals

	Total cohort (*n* = 560)	Hospital I (*n* = 311)	Hospital II (*n* = 249)	*p*-value
Age	57 (38–71)	57 (37–71)	58 (38.5–70)	0.588
Females	207 (37%)	108 (34.7%)	99 (39.8%)	0.220
Mechanism of injury				
Road traffic accident	246 (43.9%)	128 (41.2%)	118 (47.4%)	< 0.001
Fall from stairs	112 (20.0%)	57 (18.3%)	55 (22.1%)	
High energy fall	62 (11.1%)	46 (14.8%)	16 (6.4%)	
Low energy fall	52 (9.3%)	27 (8.7%)	25 (10.0%)	
Non-accidental trauma	44 (7.9%)	33 (10.6%)	11 (4.4%)	
Collapse	23 (4.1%)	14 (4.5%)	9 (3.6%)	
Other	21 (3.8%)	6 (1.9%)	15 (6.0%)	
ISS	24 (17–24)	25 (20–27)	21 (17–26)	< 0.001
Hospital LOS	12 (5–24)	11 (5–22)	13 (6–26)	0.154
ICU LOS	4 (2–9)	4 (2–11)	4 (2–8)	0.305
In-hospital mortality	158 (28.2%)	86 (27.7%)	72 (28.9%)	0.741

Data are shown as median (P_25_-P_75_) or as *n* (%)

*ICU* intensive care unit, *ISS* injury severity score, *LOS* length of stay

### Injury coding

The percentage of MAIS ≥ 4 and major trauma (ISS > 15) was higher when TBI was AIS codes were derived from the radiologic template compared with re-coded without the template (*n* = 456 (81.4%) and *n* = 374 (66.8%), *p* < 0.001; *n* = 441 (78.8%) and *n* = 352 (62.9%), *p* < 0.001, respectively). The derived ISS was not significantly different when TBI was AIS coded based on the radiologic template compared with recoding without the template (21 P_25_-P_75_ 14–26 and 21 P_25_-P_75_ 17–26). There was a significant inter-centre difference regarding injury coding of MAIS head ≥ 4 when injuries were re-coded without a template compared with coding based on the radiologic template (*n* = 212 (68.2%) and *n* = 140 (56.2%); *p* = 0.004; Table 2). This difference was not significant when injuries were coded according to the radiologic template (*n* = 251 (80.7%) and *n* = 190 (76.3%); *p* = 0.206).

**Table 2:** Differences in injury coding following the AIS by recoders and according to a standardised radiologic template, for the total cohort and both participating hospitals

	Injury coding	Total cohort (*n* = 560)	Hospital I (*n* = 311)	Hospital II (*n* = 249)	*p*-value
MAIS ≥ 4	Recoding	352 (62.9%)	212 (68.2%)	140 (56.2%)	0.004
	Template	441 (78.8%)	251 (80.7%)	190 (76.3%)	0.206
ISS	Recoding	21 (14–26)	24 (16–26)	17 (12–26)	< 0.001
	Template	21 (17–26)	24 (17–26)	20 (16–26)	0.011
MT	Recoding	374 (66.8%)	218 (70.1%)	156 (62.7%)	0.063
	Template	456 (81.4%)	257 (82.6%)	199 (79.9%)	0.411
AIS (*n*)	Recoding	5 (3–6)	5 (4–7)	4 (3–5)	< 0.001
	Template	5 (3–7)	5 (4–7)	5 (3–7)	0.906
NFS no.	Recoding	0 (0–1)	0 (0–0)	1 (0–2)	< 0.001

Data shown as median (P_25_-P_75_) or as *n* (%)

*AIS* Abbreviated Injury Scale, *ISS* injury severity score, *MAIS* Maximum Abbreviated Injury Scale, *MT* major trauma, *NFS* not further specified

The ISS remained significantly different between centres when derived from re-coded AIS TBI codes (24 P_25_-P_75_ 16–26 and 17 P_25_-P_75_ 12–26; *p* < 0.001) compared with those derived from coded injuries based on the radiologic template (24 P_25_-P_75_ 17–26 and 20 P_25_-P_75_ 16–26; *p* = 0.011) as shown in Table 2.

The coding variability did not affect the frequency of major trauma patients (ISS > 15) based on AIS head recoding by coders (*n* = 218 (70.1%) vs. *n* = 156 (62.7%); *p* = 0.063) or based on the radiologic template (*n* = 257 (82.6%) vs. *n* = 199 (79.9%); *p* = 0.411) between hospitals (Table 2). However, the number of major trauma patients was higher with the radiologic template than without it for the total cohort (*n* = 456 (81.4%) vs. *n* = 374 (66.8%), *p*-value < 0.001) and for both hospitals separately (*n* = 257 (82.6%) vs. *n* = 218 (70.1%), *p*-value < 0.001; *n* = 199 (79.9%) vs. *n* = 156 (62.7%), *p*-value < 0.001).

The frequency of AIS codes per patient based on recoding AIS head without the radiologic template differed significantly between the two centres (5 P_25_-P_75_ 4–7 and 4 P_25_-P_75_ 3–5; *p* = < 0.001). This was, however, not significantly different when injuries were re-coded according to the radiologic template (5 P_25_-P_75_ 4–7 and 5 P_25_-P_75_ 3–7; *p* = 0.906; Table 2). Finally, the number of ‘not further specified’ codes was significantly different between the two participating centres (0 P_25_-P_75_ 0–0 and 1 P_25_-P_75_ 0–2; *p* = < 0.001; Table 2).

### Reliability

Contingency tables of MAIS < 4 and MAIS ≥ 4 displayed a true positive rate of 67% and a true negative rate of 71%. The false positive rate and false negative rate were 33% and 29%, respectively, resulting in a significant difference according to McNemar’s test for the total cohort (*p* < 0.001, Table 3). The true positive rate and true negative rate were 65% and 76% for hospital I and 69% and 64% for hospital II, respectively. The false positive rate and false negative rate were respectively 35% and 24% for hospital I, and 31% and 36% for hospital II, resulting in a significant difference for both hospitals according to McNemar’s test (*p* < 0.001).

**Table 3:** Contingency tables of MAIS 3 and MAIS ≥ 4 following AIS coding by recoding without and with a radiologic template, for the total cohort and both participating hospitals

Total cohort		Template					
		MAIS = 3	MAIS ≥ 4	Total	FPR	FNR	McNemar
**Recoding**	**MAIS** = **3**	80 (14.3%)	128 (22.9%)	208 (37.1%)	33%	29%	< 0.001
	**MAIS** ≥ **4**	39 (7.0%)	313 (55.9%)	352 (62.9%)			
	**Total**	119 (21.2%)	441 (78.8%)	560			
**Hospital I**		**Template**					
		**MAIS** = **3**	**MAIS** ≥ **4**	**Total**	**FPR**	**FNR**	**McNemar**
**Recoding**	**MAIS** = **3**	39 (12.5%)	60 (19.3%)	99 (31.8%)	35%	24%	< 0.001
	**MAIS** ≥ **4**	21 (6.8%)	191 (61.4%)	212 (68.2%)			
	**Total**	60 (19.3%)	251 (80.7%)	311			
**Hospital II**		**Template**					
		**MAIS** = **3**	**MAIS** ≥ **4**	**Total**	**FPR**	**FNR**	**McNemar**
**Recoding**	**MAIS** = **3**	41 (16.5%)	68 (27.3%)	109 (43.8%)	31%	36%	< 0.001
	**MAIS** ≥ **4**	18 (7.2%)	122 (49.0%)	140 (56.2%)			
	**Total**	59 (23.7%)	190 (76.3%)	249			

*FNR* false negative rate, *FPR* false positive rate, *MAIS* Maximum Abbreviated Injury Scale

**Fig. 3:**
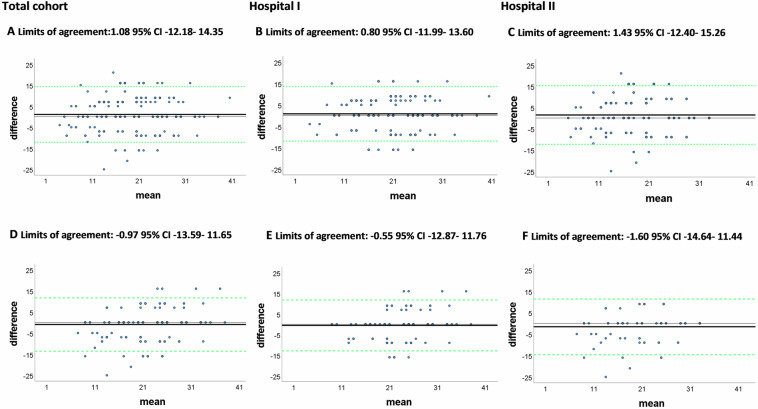
Bland–Altman plots comparing ISS based on TBI recoding without and with a radiologic template and recoding with a radiologic template compared with DNTR extracted TBI codes, for the total cohort and both hospitals separately. The first row of **A**–**C** is a comparison of injury coding based on a standardised radiologic template and injury coding without a standardised radiologic template. The second row of **D**–**F** is a comparison of injury coding based on a standardised radiologic template and injury coding extracted from the DNTR. The black line represents the mean difference between the two displayed ISS calculations following a two specific method of injury coding. The green lines represent the 95% CI limits of agreement from the mean differences

The Intraclass Correlation Coefficient (ICC) of the ISS derived from the recoding process compared with ISS derived from the radiology template was moderate for the total cohort and for both hospitals (0.73 95% CI 0.68–0.77; 0.73 95% CI 0.66–0.78; 0.77 95% CI 0.64–0.78, respectively, Fig. 3). The Bland–Altman plots indicate there is no systematic difference between the two coding methods (Fig. 3).

## Discussion

This two-centre retrospective cohort study assessed the impact of a standardised radiologic template on the injury severity assessment and classification of TBI according to the AIS of the head in two major level 1 trauma centres. The use of the radiologic template changed the classification of traumatic brain injury severity in 30% of the cases. Using the template reduced the number of missed TBI, more detailed injury codes, increased the number of cases classified as ‘major trauma’, and increased the inter-centre coding consistency. Coders differed across centres in TBI severity, major trauma classification, and number of TBI and non-specified TBI codes. Using a standardised radiologic template for injury coding evened out these differences.

Prior studies have shown that injury coding with AIS is not flawless. Classifying major trauma seems to be difficult for benchmarking due to unreliable injury coding since certified coders can miss up to 30% of injuries, of which TBI is most common [19]. However, a comparative study has shown a high rate of inter-observer reliability in a group of coders who are employees of the same institution [21]. In addition, an international study on injury coding showed high reliability within a trauma centre, but significant variability between trauma centres from different countries [25]. Previously mentioned studies show that inter-observer agreement is high when the coding process is either done by clinicians or by qualified personnel as long as the information is recorded with sufficient detail [24]. Radiologists code more consistently across trauma populations since they are trained to interpret diagnostic images. Radiologists are also used to reporting uniformly; therefore a radiologic template can help to make a translation to injury coding by coders. Even though Fig. 1 displays ostensibly that a standardised radiologic report is longer, word counts of the written parts of the two reports are nearly the same. However, the summary is longer and more specific in a standardised radiologic template compared with the summary that was not based on a standardised template. The current study shows that a standardised radiologic template is an efficient way of creating disambiguate injury reporting, which can also be implemented across different facilities. This may improve injury coding quality for both patients and populations, resulting in more reliable research and benchmarking data.

Hospital I coded the independent injuries of patients admitted to hospital II less severely, with more NFS codes and a lower ISS, than the original coders of hospital II. This is indicative of more conservative injury coding in hospital I. This is also affirmed by the frequency of patients classified as major trauma when comparing injury coding following the radiologic template than recoding without. With the standardised radiologic template as a leading method for TBI coding, the total cohort would have been classified with 22.9% more MAIS head ≥ 4 and 7.0% less MAIS head ≥ 4, resulting in 14.6% more patients classified as ‘major trauma’. This would have been 19.3% and 6.8% for hospital I, and 27.3% and 7.2% for hospital II, resulting in 12.5% and 17.2% more patients classified as major trauma, respectively. The percentage of MAIS head downgraded from MAIS ≥ 4 to MAIS head 3 is lower and similar between participating hospitals, compared with the percentage MAIS head 3 that was upgraded to MAIS head ≥ 4, which is higher in the study cohort admitted to hospital II. With major trauma quality indicators in the Netherlands currently focusing on volume and percentage directly admitted to a regional level I trauma centre, the results in this study could shed new light on the status quo of these indicators.

### Limitations and strength

A previous study showed case-mix differences between trauma populations of two hospitals [25]. AIS coding differences between the original coders might have also been present. The current study did not compensate for these differences in the analysis. Despite these differences, injury severity based on the radiologic standardised template was stable between the two hospitals. Case-mix differences across hospitals can be dependent on the local health care context, therefore extrapolating the current result (internationally) should be taken with caution.

Reassessments of injury reporting with the standardised radiologic template by the radiologists were done without time pressure, unlike the original radiologic reports without the standardised radiologic template in the acute health care setting. Also, the radiologist could focus on TBI reporting only, unlike the acute health care setting during which more variation of diseases and injury coding takes place. The radiologists were also aware of the task; this may have influenced the results towards a larger difference between the two types of TBI coding assessments. On the other hand, the two coders were very experienced, which does not explain the large difference between the two types of TBI coding assessments, especially the inter-centre difference.

There are two factors that likely underestimate the positive effect of introducing a radiologic template for TBI coding. First, only level I trauma centres participated in this study, which are also 24/7 neurosurgical facilities. Although less frequently, due to undertriage, major trauma patients are also admitted to non-level I trauma centres and a large proportion of these patients suffer from TBI [33]. Second, some specific brain injuries influencing the injury severity that are partially AIS coded by clinical symptoms were not part of the standardised radiologic template (diffuse axonal injuries and coma). Moreover, the severity of base and vault fractures could not be specified in the radiologic template. With these missing injury coding elements, coding has been relatively conservative following the standardised radiologic template, and the severity of TBI would have been higher in certain cases when such information would have been reported [27].

### Future perspectives

For an optimal effect of implementing a radiologic template some elements need to be in place. In the first place, perseverance is needed for the whole radiologic team consistently reporting CT scans according to the template. Each medical discipline that uses radiologic reports based on a standardised template should keep its motivation high on demanding consistent reporting. Second, the template and AIS should be implemented in a curriculum framework for medical students. Third, the template can be used as a basis for artificial intelligence techniques deriving AIS code from radiologic images and accessory reports. Fourth, a standardised template for other (complex) body regions might be complementary to the TBI template. Especially traumatic spine injuries and solid organ injuries (thoracic and abdominal), which have severe consequences for trauma patients and result in a high a priori mortality risk. Experience from implementing a template used for this study will likely result in improved revisions. Templates for other body regions will also benefit from these experiences. Finally, once a template is implemented, and coders more uniformly code injuries, high-quality injury coding will lead to registrations with better classification of trauma populations following their injuries. Benchmarking and financial administration can benefit from such development.

Other medical disciplines will most likely also benefit from clear communication between radiologic reports implementing reports based on specific disease classifications in specific acute health care domains. Patient outcome measures, decision making on interventions, and a minimisation of redundant financial costs could also be part of a beneficial effect that radiologic reports adapted to specific disease or injury classifications have.

## Conclusion

Coding traumatic brain injuries with the Abbreviated Injury Scale based on a structured radiologic template results in fewer missed traumatic brain injury codes, a reduction of non-specified traumatic brain injuries codes, more patients being classified as major trauma and an increase of inter-centre uniformity in coding traumatic brain injuries. A template for radiologic reporting traumatic brain injuries may positively contribute to the quality of injury coding, inter-observer reliability, reduce registration load, benchmarking, and financial administration. This could ultimately lead to increased accuracy in patient selection and subset outcome analysis in trauma populations. High-quality injury coding will improve classification of trauma populations following their injuries.

## Supplementary information

ELECTRONIC SUPPLEMENTARY MATERIAL

## Abbreviations

AIS: Abbreviated Injury Scale
CI: Confidence interval
CT: Computed tomography
DNTR: Dutch Nationwide Trauma Registry
EHR: Electronic Health Record
ER: Emergency room
ICC: Intraclass Correlation Coefficient
ICU: Intensive care unit
ISS: Injury Severity Score
MAIS: Maximum Abbreviated Injury Scale
NFS: Not further specified
TBI: Traumatic brain injury
