# Correction: Coding traumatic brain injury with the abbreviated injury scale following a standardised radiologic template will improve classification of trauma populations

**DOI:** 10.1007/s00330-026-12491-x

**Published:** 2026-03-24

**Authors:** Jan C. van Ditshuizen, Menco J. S. Niemeyer, Esther M. M. Van Lieshout, Dennis Den Hartog, Jacob J. Visser, Karlijn J. P. van Wessem, Michiel H. J. Verhofstad

**Affiliations:** 1 Trauma Research Unit, Department of Surgery, Erasmus MC, University Medical Center Rotterdam, Rotterdam, The Netherlands; 2 Trauma Centre Southwest Netherlands, Erasmus MC, University Medical Center Rotterdam, Rotterdam, The Netherlands; 3 Department of Trauma Surgery, University Medical Center Utrecht, Utrecht, The Netherlands; 4 Department of Radiology & Nuclear Medicine, Erasmus MC, University Medical Center Rotterdam, Rotterdam, The Netherlands

**Correction to: European Radiology**

10.1007/s00330-025-11384-9, published online 31 January 2025

In this article, the author’s name, Jacob J. Visser, was incorrectly written as Jan-Jaap Visser.

The original article has been corrected.

