# Supplementary material for: Coding traumatic brain injury with the abbreviated injury scale following a standardised radiologic template will improve classification of trauma populations
Source: Eur Radiol. 2025 Jan 31;35(8):5081–90. doi: 10.1007/s00330-025-11384-9 (PMC12226622; doi:10.1007/s00330-025-11384-9)
Supplement: Supplementary file 1 — ELECTRONIC SUPPLEMENTARY MATERIAL [file 330_2025_11384_MOESM1_ESM.pdf]

Coding traumatic brain injury with the abbreviated injury scale following a standardised radiologic template will improve classification of trauma populations

ELECTRONIC SUPPLEMENTARY MATERIAL

Supplemental Figure 1. Standardised radiologic template

Clinical data

Examination question:

Comparative previous examinations: [...]

Description:
-Protocol

-Extracranial injuries: [...]
-Base fracture: [...]
-Vault fracture: [...]
-Panfacial fracture: [...]
-Pneumocephalus: [...]
-Subarachnoid hemorrhage: [...]
-Extra-axial hematoma (including coronal-plain max. diameter in mm.): [...]
-Intracerebral hematoma, including contusion (max. length in mm.): [...]
-Petechial hemorrhage(s): [...]
-Midline shift (mm.): [...]
-Herniation: [...]
-Ventricle(s): [...]
-Compressed brain stem cisterns: [...]
-Other findings: [...]

Summary:

Conclusion discussed with [...] at [...] on [...]
